# Neuroinflammation in a Rat Model of Tourette Syndrome

**DOI:** 10.3389/fnbeh.2022.710116

**Published:** 2022-03-10

**Authors:** Ke Zhongling, Chen Yanhui, Chen Guofeng, Liu Yanyan

**Affiliations:** Fujian Medical University Union Hospital, Fuzhou, China

**Keywords:** Tourette syndrome, neuroinflammation, cytokines, microglia, IDPN

## Abstract

**Objective:**

Tourette syndrome (TS) is a group of childhood-onset chronic neuropsychiatric disorders characterized by tics, i.e., repetitive, sudden, and involuntary movements or vocalizations, which is often associated with various psychopathological and/or behavioral comorbidities, including attention deficit hyperactivity disorder (ADHD), obsessive-compulsive disorder (OCD), anxiety, depression, and sleep disorders and have a worse prognosis. The mechanism of TS is still not clear. The relationship between immune activation, neuroinflammation, and neuropsychiatric disorders has attracted much attention in the past two decades. To explore the underlying mechanism in TS, the relationship between neuroinflammation and behavioral alterations in TS rats was investigated in this study.

**Methods:**

A total of 36 Sprague-Dawley male rats were divided into three groups randomly as follows: the TS, control (CON), and drug intervention groups. The TS rat group was treated with haloperidol (Hal) (the TS + Hal group). The TS rat model was established using 3,3-iminodipropionitrile (IDPN), which is a well-known animal model of TS. The behavioral syndromes, brain tissue cytokines, like interleukin (IL)-6 and tumor necrosis factor-alpha (TNF-α), and microglial activation of the three groups were assessed.

**Results:**

The behavioral scores of rats in the TS group and the TS + Hal group were higher than those in the CON group (*P* < 0.05), but the scores of behavioral tests in the TS + Hal group were lower than those in the TS group (*P* < 0.05). The levels of IL-6 and TNF-α in the rat brain tissue were significantly higher in the TS group than in the CON group (*P* < 0.05), while no significant differences were found between the CON group and the TS + Hal group (*P* > 0.05). The microglia was significantly activated in the TS group and slightly activated in the TS + Hal group, which was considerably less than that in the TS group.

**Conclusion:**

The IDPN-induced TS rats had significant neuroinflammation in the brain, and the interaction between dopamine (DA) dysregulation and immune dysfunction may play a vital role in the pathogenic mechanisms of TS.

## Introduction

Tic disorders are common neurodevelopmental disorders in children, often begin between the ages of 4 and 8 years, and are more common in boys than in girls (3:1–4:1); the main clinical manifestations are involuntary, repetitive, rapid, purposeless motor or vocal tics in one or more areas, which can be accompanied by behavioral problems and neuropsychological difficulties (Singer, 2019). Tourette syndrome (TS) is the most severe type of tic disorder, characterized by multiple motor tics and vocal tics that have been present for more than 1 year (Coffey, 2020), which significantly impact children’s social and academic activities. The etiology and pathogenesis of tic disorders have not yet been elucidated.

The relationship between immune activation, neuroinflammation, and behavioral symptoms had been largely studied in the past two decades, especially in the pediatric autoimmune neuropsychiatric disorder associated with streptococcus (PANDAS)/pediatric acute-onset neuropsychiatric syndrome (PANS; Murphy et al., 2010; Swedo et al., 2015, 2017; Zheng et al., 2020; Murgia et al., 2021). Some TS cases, considered as a putative example of such phenomenon, meet the criteria for PANDAS/PANS, suggesting that the two diseases may have similar etiological mechanisms in some specific situations (Lamothe et al., 2021). Thus, it is reasonable to assume that TS also involves immune processes. Some studies have shown that various etiologies can lead to the development of TD by affecting the immune system (Martino et al., 2015). Li et al. (2015) found that interleukin (IL)-6 and IL-8 levels in patients with TS were lower than those in the normal group. Morer et al. (2010) showed a significant increase in gene expression encoding IL-2 and IL-2 receptor beta in patients with TS. In addition, a case-control study, including 117 patients with TS found that the frequency of the tumor necrosis factor (TNF)-308G allele, was higher in children with TS than in controls (90.2 vs. 84.8%, *P* = 0.037) (Keszler et al., 2014). Although these studies have suggested there were immune disorders in patients with TS, the specific mechanism between neuroinflammation and symptoms in patients with TS is still not clear. 3,3-Iminodipropionitrile(IDPN) can induce dysregulation of dopamine (DA) in rat brain, which reproduces the behavioral characteristics of TS comprehensively, and it is easy to establish with a long duration of tic symptoms, which is a well-known animal model of TS (Yanhui, 2020). In this study, we used IDPN to construct the TS rat model to explore the relationship between neuroinflammation and behavioral alterations and to explore the underlying mechanisms in TS.

## Materials and Methods

### Experimental Animals

A total of 36 postnatal 21 days old (P21) Sprague-Dawley (SD) male rats (weighing 60–70 g) were purchased from Fuzhou Wu Laboratory Animal Center, were housed in separate cages at constant temperature and humidity (temperature: 22 ± 2°C and humidity: 50 ± 10%), and were fed and watered freely.

### Methods

#### Animal Grouping and Tourette Syndrome Animal Model Establishment

A total of 36 SD rats were randomly divided into the normal control group (CON group) (*n* = 12) and the TS model group (*n* = 24). Rats in the CON group were injected intraperitoneally (i.p.) with normal saline (1 ml/kg/day). The TS model was established by i.p. injection of IDPN (TCI Shanghai Co., Ltd., Shanghai, China) at a dose of 150 mg/kg/day in a volume of 1 ml/kg for 7 consecutive days. Subsequently, the rats in the TS group were further assigned randomly to the TS group (*n* = 12) and the TS + haloperidol group (TS + Hal group) (n = 12).

#### Drug Treatment

The rats in the TS + Hal group were administered with Hal (Shanghai Xinyi Jiufu Pharmaceutical Co., Ltd., Shanghai, China) at a dose of 0.5 mg/kg/day in a volume of 0.1 mg/ml diluted with saline for 7 days, while the rats in the CON and TS groups were given an equal volume of saline.

#### Behavioral Assessment

After 7 days of drug intervention, videos were taken three times a day, 5 min each time, respectively, at 8:00 a.m., 2:00 p.m., and 8:00 p.m., then two trained observers separately and independently evaluated the stereotypical behaviors of the rats in each group and calculated the average scores of the behavior at the three time periods. The average score of their assessment was used as the final result. The animal behaviors of TS were determined by all three validity criteria, such as tics, stereotypies were repetitive, and habit-forming motor patterns, which typically mimic purposeful behaviors (Godar et al., 2014). Thus, continuous circling behavior and increased orofacial and head-bobbing behaviors in rats were considered as stereotypies. Scoring criteria for stereotypical behaviors are shown in the following table (Lin et al., 2019):

**Table:** 

Scores	Stereotypies
0	No stereotypy or normal activity
1	Discontinuous circling behavior (clockwise/counterclockwise circling) Occasional head twitching
2	Occasionally vertical dyskinetic head and neck movements Occasional sniffing, licking, and biting
3	Continuous circling behavior, increased body raising Increased sniffing, repetitive grooming (such as paw-to-mouth movements)
4	Increased lateral and vertical dyskinetic head and neck movements

#### Rat Brain Tissue Specimen Collection

After the behavioral assessment, the rats were weighed and anesthetized by i.p. injection of 10% chloral hydrate (0.03 ml/kg) and placed on the operation table, lying on its back. The skin, abdominal cavity, and diaphragm were opened in turn. Then, we cut off the chest cavity along with the rib cartilage and fixed sternum and both sides of muscles using vessel forceps to expose the heart. A hypodermic needle was inserted into the left ventricle and kept it from moving, and auricula dextra was opened using a small pair of scissors. The heart was flushed quickly with 100 ml normal saline until the effluent was essentially transparent, then rats were decapitated and the brains were dissected out quickly. The left side of the brain was placed in an Eppendorf tube and stored in a refrigerator at −80°C for ELISA; the right side of the brain was fixed in 4% paraformaldehyde immediately and was routinely paraffin-embedded and sectioned in 5 μm for immunohistochemistry.

#### Detection of Inflammatory Cytokine Levels

The levels of IL-6 and TNF-α in the rat hemicerebrum were detected using ELISA kits (Boster Biological Technology Co., Ltd., Wuhan, China), according to the manufacturer’s instructions.

#### Analysis of Active Microglia

Ionized calcium-binding adapter molecule 1 (1Iba-1) is a surface antibody that is lowly expressed in the resting state of microglia and highly expressed in activated microglia. So the expression of Iba-1 in the brain can reflect whether the microglia is activated or not. The brain sections were placed in xylene I for 15 min, xylene II for 15 min, anhydrous ethanol I for 5 min, anhydrous ethanol II for 5 min, 95% alcohol I for 5 min, 95% alcohol II for 5 min, 90% alcohol I for 5 min, 80% alcohol I for 5 min, and 70% alcohol for 5 min, then, the sections were washed in a 0.1 M phosphate buffer solution (PBS) for 5 min each. Sections were then placed in the citric acid antigen repair buffer (pH: 6.0). Antigen repair was carried out in a microwave oven, with medium temperature for 8 min, ceasefire for 8 min, and low temperature for 7 min. After natural cooling, the sections were rinsed in PBS and blocked with 3% bovine serum albumin (BSA) for 30 min and then incubated with rabbit polyclonal anti-Iba1 (dilution: 1:800) (Proteintech Co., Ltd., Wuhan, China) at 4°C overnight. Next, the sections were washed in PBS three times for 5 min each and were then incubated with goat anti-rabbit antibody IgG (dilution: 1:200) (Servicebio Co., Ltd., Wuhan, China) for 30 min at 37°C, followed by rinsing in PBS and colorimetric development (DAB) (Maixin Biotechnologies Co., Ltd., Fuzhou, China). Then, the sections were incubated with hematoxylin for the cell nucleus staining for 20 s. Sections were washed three times in PBS for 5 min and then coverslipped. Immunohistochemical paraffin sections of each group were observed under an orthomosaic biomicroscope. Three files of views in the striatal region were randomly selected for each section under a 40 × 10 microscope. Then, the ImageJ software was used to measure the average optical density (AOD) of each section.

#### Statistical Analysis

Data are expressed as the mean ± SEM. More than one independent constant variable with normal distribution was compared using ANOVA, followed by *post hoc* analyses using the Tukey’s HSD test, while the Kruskal–Wallis test was used for independent and non-normally distributed constant variables, followed by *post hoc* analyses using the Steel-Dwass test. All statistical analyses were performed using the R software, and *P* < 0.05 was considered statistically significant.

## Results

### Assessment of Stereotypical Behaviors

**FIGURE 1:**
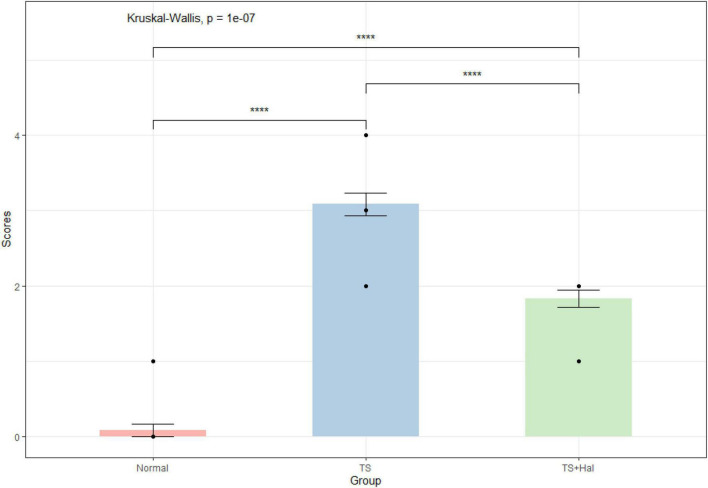
Comparison of behavioral scores in each group. TS, Tourette syndrome; TS + Hal, TS + haloperidol group;★★★★: *P* < 0.001.

There were significant differences among the CON, TS, and TS + Hal groups (*P* < 0.05), and the scores of the TS + Hal group were lower than the TS group but still higher than the CON group (Table 1 and Figure 1).

**TABLE 1:** Comparison of scores of behavior assessment in each group.

Groups	Scores	
CON group	0.08 ± 0.08	
TS group	3.08 ± 0.15	
TS + Hal group	1.83 ± 0.11	
Kruskal–Wallis chi-squared	32.21	
df	2	
*P*-value	1.01e-07	

**Steel-Dwass test**	***t*-value**	***P*-value**

CON:TS	4.50	1.98e-05
CON:TS + Hal	4.50	2.05e-05
TS:TS + Hal	4.19	8.18e-05

*CON, normal control; TS, Tourette syndrome; TS + Hal, TS + haloperidol.*

### Levels of IL-6 and TNF-α in Brain Tissue

**FIGURE 2:**
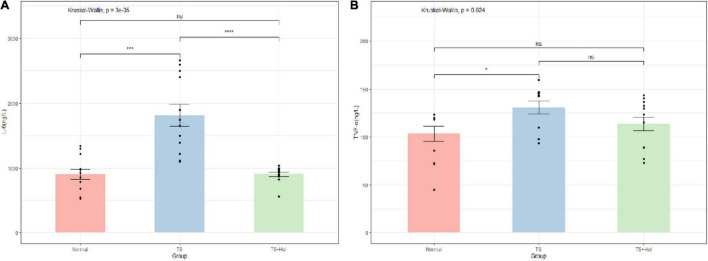
**(A)** Comparison of IL-6 levels in the brain of each group; **(B)** comparison of TNF-α levels in the brain of each group. TS, Tourette syndrome; TS + Hal, TS + haloperidol group; ns, not significant;★★★★: *P* < 0.0001; ★★★: *P* < 0.001; ★:*P* < 0.1.

The levels of IL-6 and TNF-α in brain tissue were significantly higher in the TS group than in the CON group (*P* < 0.05), while no significant differences were found between the CON and TS + Hal groups (*P* > 0.05) (Table 2 and Figure 2).

**TABLE 2:** Comparison of IL-6 and TNF-α levels in each group.

Groups	IL-6 (ng/L)	TNF-α (ng/L)
CON group	901.17 ± 79.10	103.49 ± 7.86
TS group	1812.25 ± 169.98	130.82 ± 6.80
TS + Hal group	904.92 ± 35.78	113.61 ± 7.29
Kruskal–Wallis chi-squared	20.85	7.4
df	2	2
*P*-value	2.96e-05	0.024

**Steel-Dwass test**	***t*-value**	***P*-value**

**IL-6**		
CON:TS	3.70	6.42e-04
CON:TS + Hal	0.43	9.02e-01
TS:TS + Hal	4.16	9.56e-05
**TNF-α**		
CON:TS	2.31	0.05
CON:TS + Hal	1.19	0.46
TS:TS + Hal	2.14	0.08

*CON, normal control; TS, Tourette syndrome; TS + Hal, TS + haloperidol.*

### Microglial Activation in the Striatal Area

**FIGURE 3:**
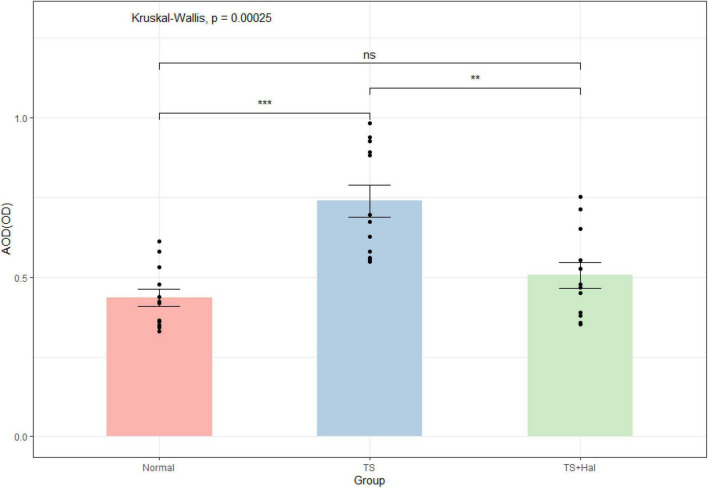
The comparison of activated microglia (AOD) in the brain of each group. TS, Tourette syndrome; TS + Hal, TS + haloperidol; ns, not significant;★★★ : *P* < 0.001; ★★: *P* < 0.01.

**FIGURE 4:**
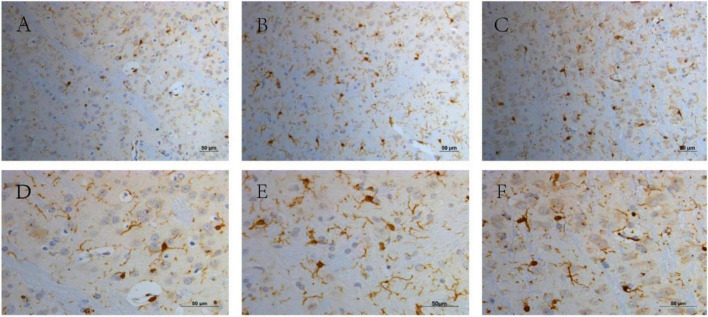
The activated microglia in the striatal area in each group. **(A)** The CON group (200×); **(B)** the TS group (200×); **(C)** the TS + Hal group (200×); **(D)** the CON group (400×); **(E)** the TS group (400×) and **(F)** the TS + Hal group (400×). CON, normal control; TS, Tourette syndrome; TS + Hal, TS + haloperidol.

Microglia were significantly activated in the TS group, and there were only very few activated microglia in the CON group. Microglial activation in the TS + Hal group was considerably lower than that in the TS group but slightly higher than that in the CON group. However, the AOD between the TS + Hal and CON groups was not statistically significant (Table 3 and Figures 3, 4).

**TABLE 3:** Comparison of AOD of active microglia in each group.

Groups	AOD of active microglia	
CON group	0.44 ± 0.03	
TS group	0.74 ± 0.05	
TS + Hal group	0.51 ± 0.04	
Kruskal–Wallis chi-squared	16.57	
df	2	
*P*-value	0.00025	

**Steel-Dwass test**	***t*-value**	***P*-value**

CON:TS	3.72	0.00
CON:TS + Hal	1.33	0.38
TS:TS + Hal	3.00	0.01

*CON, normal control; TS, Tourette syndrome; TS + Hal, TS + haloperidol; AOD, average optical density.*

## Discussion

In this study, we revealed alteration of cytokine (IL-6 and TNF-α) expressions in the hemicerebrum and significantly active microglia in the striatum in IDPN-treated rats, and Hal treatment significantly prevented the behavioral stereotypies as well as the increased cytokines expression and striatal active microglia in those IDPN-induced TS rats. All those findings suggested that immune mechanisms might be involved in the pathophysiology of TS.

The IDPN model was first used by Diamond (1982) to develop the TD model. It disrupts the DA in the extrapyramidal system, resulting in a persistent decrease in DA concentration (Nobuo Wakata, 2000), leading to DA receptor hypersensitivity during growth and development, which could result in stereotypical behaviors in animals (Yanhui, 2020). The IDPN model is a standard animal model for TS, which can reproduce the behavioral characteristics of TS comprehensively; it had been used in many experiments of TS (Zhang and Li, 2015; Zhao et al., 2015; Lin et al., 2019; Liu et al., 2020).

Although tic disorders are a prevalent developmental disease in children, the etiology and pathogenesis of tic disorders are still unclear. The central hypothesis of its pathogenesis is the cortico-striatal-thalamo-cortical (CSTC) abnormal anatomical pathways and alterations in the neurotransmitters located within these circuits (Augustine and Singer, 2018). Among the possible pathogenic mechanisms of TS, the evidence supporting dopaminergic abnormalities in TS is extensive, including the recognition that the most effective inhibitory drugs are DA receptor antagonists, such as Hal, which are widely used in the treatment of TS (Augustine and Singer, 2018). In recent decades, there were growing evidence of immunity involvement to TS, but the mechanism is still not clear (Hsu et al., 2021).

Cytokines, an important mediator in cell communication, play an important role in neuroinflammation (Becher et al., 2017), and it was also noticed in patients with TS. Yeon et al. (2017) found that compared with the healthy CON group, the serum IL-17a, IL-12p70, IL-6, and TNF-α of children with TS were significantly increased. Parker-Athill et al. (2015) also found that the serum IL-4, IL-8, and IL-17 of children with tic symptoms were considerably higher than those of normal children. The study by Liu et al. (2020), like ours, used IDPN to construct an animal model of TS, which found that plasma IL-4, IL-10, IL-12, IFN-γ, and TNF-α levels were significantly increased in TS rats, while TGF-β levels were not entirely different; Liu’s study involved mainly the immune response in the periphery of TS rats, while our study focused on the inflammation of the central nervous system (CNS) in TS rats.

Our study showed that the rat model of TS with DA dysregulation constructed using IDPN had significantly higher cytokines like IL-6 and TNF-α in the brain than in the normal group. The present data confirmed and extended prior findings that TS rats were associated with increased cytokines expression (Long et al., 2019b; Liu et al., 2020). Long et al. (2019a) also found highly elevated cytokines in a TS model constructed using the 5-hydroxytryptamine agonist 1-(2,5-dimethoxy-4-iodophenyl)-2-aminopropane (DOI). They discovered that rhynchophylline can inhibit the inflammatory response by acting on the brain-derived neurotrophic factor (BDNF)/nuclear factor-κB (NF-κB) pathway, which then improved TS symptoms. In addition, several studies have reported that passive transfer of cytokines to animals leads to the development of behavioral and neurochemical abnormalities similar to tic disorders (Hornig and Lipkin, 2013). These results support the existence of a link between immunity dysfunction and behavioral stereotypes. The TS animal models [ours and previous studies (Long et al., 2019a,b; Liu et al., 2020)] constructed by neurotransmitter dysfunction showed increased cytokines, which may also indicate an interaction between neurotransmitters and neuroinflammation in TS disorders. Animal models constructed by IDPN mainly lead to a decrease in DA neurotransmission (Nobuo Wakata, 2000), and some studies have found that decreased CNS DA leads to increased neurological inflammation (Pajares et al., 2020). This may explain the abnormalities of cytokines in TS rats in this study. In addition, cytokines, in turn, can reduce the availability of DA by increasing the expression and function of presynaptic reuptake pumps for DA and by decreasing enzymatic cofactors, such as tetrahydrobiopterin (BH4), which is a rate-limiting enzyme of DA synthesis (Miller and Raison, 2016). The interaction between neurotransmitters and neuroinflammation may be one of the crucial mechanisms in the pathogenesis or exacerbation of TS.

Microglia is the innate immune system of the CNS, and microglial activation is closely associated with CNS inflammation (Abe et al., 2020). The abnormal activation of microglia may be one of the critical factors in the neuroinflammation of TS. For patients with TS, it was found that the microglia were activated. Lennington et al. (2016) compared the autopsy results of brain tissue from nine patients with TS with nine normal subjects. They found significantly elevated expression of genes regulating microglial activation in patients with TS, as well as extremely high CD45+ cells in the striatal region of TS and microglial activation. In line with this report, using PET scanning to evaluate neuroinflammation in the brain, Kumar et al. (2015) suggested activated microglia-mediated neuroinflammation in bilateral caudate nuclei in children with PANDAS and TS. Our study also found that compared with the CON group, the microglia in the striatum of TS rats were significantly activated, which was consistent with previous studies of patients with TS (Kumar et al., 2015; Lennington et al., 2016). Active microglia can produce proinflammatory cytokines, such as IL-6 and TNF-α (Kwon and Koh, 2020), which was also found to increase in the TS group in our study. Increased cytokines, in turn, reduce the availability of DA resulting in DA dysfunction that may cause tic disorders. After Hal treatment, the microglia in the striatum of TS rats were still slightly activated, but it was considerably lower than the TS group. A variety of DA receptors can be expressed on the surface of microglia, which are involved in regulating neuroinflammation and affecting neuronal survival (Xia et al., 2019). DA receptor 2 is expressed only on the surface of activated microglia. The use of DA receptor 2 agonists increases LPS-induced nitrite release from activated mouse microglia, which indicated that DA receptor 2 plays a vital role in regulating neuroinflammation (Huck et al., 2015). In this study, the activation of microglia in the brain of rats, as well as the stereotypical behaviors and IL-6 levels of TS rats, were significantly reduced after treating with DA receptor 2 antagonist Hal. Hal may inhibit the activation of microglia by blocking DA receptor 2 and then reduce the release of pro-inflammatory factors, such as IL-6.

## Conclusion

The rat model of TS with DA dysregulation constructed using IDPN has significantly elevated cytokines and activated microglia in the brain, which supports the existence of immune dysfunction in TS, and the interaction between DA neurotransmitter and CNS inflammation may be one of the essential pathogenic mechanisms of TS, and microglial activation may be a key point.

## Data Availability Statement

The raw data supporting the conclusions of this article will be made available by the authors, without undue reservation.

## Ethics Statement

The animal study was reviewed and approved by the Institutional Review Board (or Ethics Committee) of Fujian Medical University.

## Author Contributions

KZ contributed significantly to arrange the experiment and analyze the data and manuscript preparation. CY contributed to the conception of the study and revised the article. CG and LY contributed to perform the experiments. All authors contributed to the article and approved the submitted version.

## Conflict of Interest

The authors declare that the research was conducted in the absence of any commercial or financial relationships that could be construed as a potential conflict of interest.

## Publisher’s Note

All claims expressed in this article are solely those of the authors and do not necessarily represent those of their affiliated organizations, or those of the publisher, the editors and the reviewers. Any product that may be evaluated in this article, or claim that may be made by its manufacturer, is not guaranteed or endorsed by the publisher.
